# Construction of a replication-defective recombinant virus and cell-based vaccine for H9N2 avian influenza virus

**DOI:** 10.1186/s13567-025-01577-x

**Published:** 2025-07-08

**Authors:** Lijin Lai, Rui Li, Yanan Chen, Junyuan Deng, Siyao Yu, Qiuyan Lin, Libin Chen, Tao Ren

**Affiliations:** 1 College of Veterinary Medicine, South China Agricultural University, Guangzhou, 510642 China; 2 National and Regional Joint Engineering Laboratory for Medicament of Zoonosis Prevention and Control, Guangzhou, China; 3 Key Laboratory of Animal Vaccine Development, Ministry of Agriculture, Guangzhou, China; 4 Key Laboratory of Zoonosis Prevention and Control of Guangdong Province, Guangzhou, China

**Keywords:** Avian influenza virus, H9N2 subtype, reverse genetics system, vaccine efficacy

## Abstract

The H9N2 subtype of avian influenza is highly contagious, and although it is classified as a low-pathogenic avian influenza virus, its tendency to recombine with other subtypes of avian influenza viruses has made it a potential problem for the poultry industry. Vaccines currently used to prevent this disease are all inactivated, making it difficult to stimulate long-lasting immunity, and have a very weak ability to trigger cellular immunity, thus failing to address the problem of virus shedding. Live-attenuated vaccines are capable of stimulating cellular immunity but carry the risk of recombination with wild-type strains. In this study, we successfully rescued a replication-deficient H9N2 strain (H9-SD18GD12HA) using reverse genetic techniques, which was obtained by replacing the neuraminidase (NA) gene with the open reading frame of the hemagglutinin (HA) gene with the PR8 strain as the backbone. Dynamic growth results showed that H9-SD18GD12HA can proliferate only under NA-containing conditions and therefore cannot grow in normal animals or cells. After immunization of chickens with H9-SD18GD12HA using eye and nose drops, both humoral and cellular immunity were stimulated, and some degree of reduction in virus shedding was observed. These results indicate that H9-SD18GD12HA has good immunogenicity, does not proliferate in vivo, and has the potential to be developed into a novel live-attenuated vaccine for the H9N2 subtype of avian influenza.

## Introduction

H9 subtype avian influenza (AI), caused by H9 subtype avian influenza virus (AIV), was first reported in China in 1992 and subsequently spread to become a widespread infectious disease throughout the country [1], with the H9N2 subtype being the main strain of avian influenza subtype H9 currently circulating globally [2]. The H9N2 subtype is a low-pathogenic AIV (LPAIV), and chickens infected with H9N2 typically do not develop the disease or show only mild symptoms, which makes long-term persistence of the virus and continuous virus shedding possible [3]. After more than 30 years of epidemics, H9N2 has not only gradually adapted to spread in chickens but some newer strains have also shown infectivity in mammals and carry the risk of triggering zoonotic epidemics [1, 4, 5]. It is worth noting that although H9N2 does not cause severe symptoms or death in chickens, it is usually able to contribute internal gene cassettes as a donor to other subtypes of influenza viruses, such as H5N1, H5N6, and H7N9 [6, 7], which undoubtedly increases the risk and difficulty of preventing and controlling H9N2 and other subtypes of AIV. In addition, the available evidence also suggests that H9N2, as the donor of the internal gene cassette, is involved in the recombination of H3, H5 and H7 subtypes of AIVs that have led to human cases of infection [8–10], further emphasising the public health importance of H9N2 AIV prevention and control.

Based on the differences in HA nucleotide sequences, H9N2 subtype AIVs have been classified into two main lineages, the Eurasian lineage and the North American lineage, of which the Eurasian lineage has been further classified into many sub-lineages, including A/chicken/Beijing/1/1994 (BJ/94-like), A/quail/Hong Kong/G1/1997 (G1-like), A/duck/ Hong Kong/Y280/1997 (Y280-like), A/duck/Hong Kong/Y439/1997 (Y439-like), and A/chicken/Shanghai/F/1998 (F/98-like) [1, 11]. In recent years, the main epidemiological strains of AIV in poultry in China are BJ/94 and F/98, whereas the main strain of AIV that infects humans is Y280 [12, 13].

The vaccines currently licensed in China for the prevention and control of avian influenza subtype H9N2 are inactivated using strains such as F/98, A/Chicken/Guangdong/SS/94 (SS/94), and A/Chicken/Henan/01/2006 (HN106) as reference strains [14]. Because the reference strains used are all earlier isolates, it would be difficult for existing vaccines to effectively prevent chickens from being infected in the context of the continuous evolution of the H9N2 subtype AIV. Previous reports have shown that inactivated vaccines using early isolates, such as F/98 and SS/94, as reference strains, although able to alleviate symptoms, were unable to stop the prevalence and spread of H9N2, and the problem of viral shedding could not be better addressed [15, 16]. In contrast, incomplete and inefficient vaccine protection may promote the adaptation and evolution of the virus [17].

In 2000, Hoffman et al. established an 8-plasmid influenza virus reverse genetics system. This system utilized a bi-directional set of expression vectors [18], incorporating an RNA polymerase II promoter and poly-A signaling in the forward direction at either end of the insertion fragment. The reverse orientation facilitated transcription of viral cDNA into vRNA by RNA polymerase I. Meanwhile, RNA polymerase II transcribed mRNA to produce viral proteins. Each plasmid enabled the replication of viral genes and the synthesis of proteins within the respective segments after cell transfection. The eight RNA fragments of the AIV are indispensable for viral proliferation, and the prevailing view is that they are specifically packaged into viral particles rather than assembled non-specifically, as suggested by the packaging-on-the-fly hypothesis [19, 20]. Fujii et al. demonstrated that the packaging signals of AIV RNA fragment 6, the segment that translates the NA protein, are not only present in the non-coding region but also extend to both ends of the coding region. Additionally, they reported that the packaging signals in the coding region at the 3ʹ end are of more value [21]. It has been shown that the functional strength and coordination of viral packaging may be related to site-specific charges of the viral nucleoprotein (NP) and RNA-RNA interactions [22]. Based on the selective-incorporation model, substitution of viral genome packaging signals may also negatively affect the efficiency of recombinant virus production or infection as well as to RNA–RNA interactions [23].

Although inactivated vaccines have the advantages of being inexpensive, easy to store, and not replicating in the body without the risk of virulence back-strengthening [24], they can usually only induce a humoral immune response in the body, making it difficult to generate cellular immunity, whereas live viruses that have been modified as antigens for immunization, through genetic engineering for instance, are capable of inducing a comprehensive and long-lasting immune response [25]. There have been many research attempts to construct virulence-reduced or reassortment-impaired viruses by reverse genetics, and takedown protection tests have been conducted, demonstrating that the strains rescued by these methods have at least a weak ability to transmit in animals or are unable to act as contributors to the reassortment of gene fragments [26–28]. The NA protein plays an important role in the life activities of the influenza virus. It is a glycoprotein on the surface of the virus that induces protective immunity against AIV. Also, NA is responsible for the cleavage of sialic acid residues during the budding process after the formation of the virus and assists in the release of the virus from the host cell. The absence of NA protein will cause a dramatic reduction in the efficiency of virus replication and the loss of its ability to continue replicating. However, there have been no previously reported attempts to construct a replication-defective vaccine by replacing or deleting NA yet. Here, we used reverse genetics to generate a recombinant H9N2 subtype AIV by replacing the NA fragment of the viruses with an HA fragment while preserving the packaging signal. Consequently, the recombinant strains lacked NA protein functionality, rendering them incapable of completing the full viral replication cycle. This design ensured biosafety and enhanced the potential of producing better humoral and cellular immunity following immunization. This study demonstrates the potential of constructing replication-defective live virus vaccines for the prevention and control of H9N2 AIV transmission and pandemics without additional biosafety concerns, particularly based on the ease of recombination in AIV.

## Materials and methods

### Ethics statement

The animal study protocol was approved by the South China Agricultural University Experimental Animal Welfare Ethics Committee (permit number: 2022F185). All animal experiments were performed in the authorized animal biosafety level 2 (ABSL 2) facilities at South China Agricultural University.

### Virus isolation and purification

Two strains of H9N2 AIV were isolated from chickens in Guangdong and Shandong provinces in China. Oropharyngeal and cloacal swabs that tested positive for AIV were thawed on ice, mixed using a vortex mixer, and inoculated into 9–11-day-old specific-pathogen-free (SPF) chicken embryos. Embyonated-eggs with embryos that died within 24 h after inoculation were removed. The allantoic fluid from the remaining embyonated-eggs was collected 96 h after inoculation for virus purification. The harvested virus-containing allantoic fluid was centrifuged at 2000 rpm for 10 min at 4 °C and then filtered using a 0.22 μm filter to obtain pure virus-containing allantoic fluid. After purification, the allantoic fluid was subjected to a hemagglutination assay to determine hemagglutination potency, aliquots were taken and stored at −80 °C for subsequent experiments.

Viral genomic RNA was extracted separately from allantoic fluid containing each of the two H9N2 virus strains using an RNAfast200 RNA Extraction Kit (Fastagen, Shanghai, China). The influenza virus reverse transcription primer Uni12 was used for reverse transcription using Reverse Transcriptase M-MLV (Takara Bio, Beijing, China), according to the manufacturer’s instructions. The cDNA obtained from reverse transcription was used as a template, and specific primers (Table 1) were used to amplify the HA and NA fragments. The amplified fragments were subjected to nucleic acid gel electrophoresis. The gel was recovered and subjected to Sanger sequencing (Sangon, Shanghai, China). Sequence splicing was performed using DNASTAR Lasergene 7. Two strains of H9N2 subtype AIV isolated from Guangdong and Shandong were confirmed and named A/chicken/Guangdong/GD12/2022 (GD12) and A/chicken/Shandong/SD18/2022 (SD18), respectively.

**Table 1:** **Primers for HA sequence amplification**

Primer name	Primer Sequences (5ʹ → 3ʹ)
AIV-HA-F	AGCRAAAGCAGGGGAATTTCACAAC
AIV-HA-R	AGTAGAAACAAGGGTGTTTTTGCCAA
AIV-NA-F	AGCRAAAGCAGGAGTAAAAATGAAT
AIV-NA-R	AGTAGAAACAAGGAGTTTTTTCTAAAA

### Cell culture and preservation

Human embryonic kidney (293T) and Madin-Darby canine kidney (MDCK) cells were cultured in Dulbecco’s modified Eagle’s medium (DMEM; Gibco, Waltham, Massachusetts, USA) with 10% fetal bovine serum (FBS; Gibco) respectively, at 37 °C with 5%. The MDCK cell line NA-MDCK, constructed using lentiviral infection and capable of expressing NA protein, was constructed and preserved in our laboratory.

### Genetic evolutionary analysis of HA gene sequences

Partial sequences of each lineage, reference strains of H9N2 inactivated vaccines available in China, and classical isolates were obtained from the Global Initiative on Sharing Avian Influenza Data (GISAID) EpiFlu database [29]. The HA sequences were used for subsequent phylogenetic analyses together with the sequences of GD12 and SD18 after removing duplicates and low-quality sequences. Phylogenetic analysis was performed with Phylosuite (v1.2.3) using MAFFT (v7.505) and Gblocks (v0.91b) for sequence comparison and trimming, and ModelFinder (v2.2.0) was used to select the best-fit model using the BIC criterion (GTR+F+I+G). Maximum likelihood phylogenies were inferred using IQ-TREE (v2.2.0), with the (GTR+F+I+G) model for 1000 standard bootstraps. The Interactive Tree of Life (iTOL) [30] was used for the visualization and beautification of phylogenetic trees.

### Plasmid construction

The NA ORF fragment was amplified using the primers pLOV-NA-F and pLOV-NA-R and viral cDNA as a template. The lentiviral expression plasmid pLOV-CMV-PuroR was amplified using the primers pLOV-F and pLOV-R to obtain a linearized vector homologous to the NA gene fragment which resulted in a recombinant plasmid pLOV-CMV-NA-PuroR.

The SD18 cDNA was used as a template to amplify the full-length HA gene using the pHW-H9HA-F and pHW-H9HA-R primers. The linearized vector was amplified using pHW2000 as the template and pHW-F and pHW-R as primers. The HA fragment of SD18 was ligated to pHW2000 via homologous recombination to obtain the recombinant plasmid pHW-SD18HA.

The cDNA of GD12 was used as a template to amplify the ORF fragment of the HA gene using HA(ORF)-F and HA(ORF)-R primers. The linearized vector containing the NA packaging signal was amplified using pHW-PR8-NA as the template and PHW-NAPS-F and PHW-NAPS-R as primers. The HA (ORF) fragment of GD12 was ligated to pHW-PR8-NA via homologous recombination to obtain the recombinant plasmid pHW-PR8NAPS- GD12HA. The primer sequences used for plasmid construction are listed in Table 2.

**Table 2:** **Primers for the construction of lentiviral packaging plasmids and reverse genetics**

Primer name	Primer Sequences (5ʹ → 3ʹ)
pLOV-NA-F	GATCCGCCACCATGAATCCAAATCAGAAGATAACAG
pLOV-NA-R	GGCGGCCGCTTATATAGGCATGAAGTTGATATTCGC
pLOV-F	GCGGCCGCCCTAGGCGTC
pLOV-R	GGTGGCGGATCCAAATCC
pHW-H9HA-F	GAAGTTGGGGGGGAGCAAAAGCAGGGGAATTTCACA
pHW-H9HA-R	GGCCGCCGGGTTATTAGTAGAAACAAGGGTGTTTTTGCC
HA(ORF)-F	AATAGAAAAGAACATGGAGACAGTATCACTAATAACTATACTA
HA(ORF)-R	CCACATAAAAACACCTGTTGTTATATACAAATGTTGC
pHW-NAPS-F	CAACAGGTGTTTTTATGTGGAGTTG
pHW-NAPS-R	GTTCTTTTCTATTATGATTGGTTCACATG
pHW-F	AATAACCCGGCGGCCCAA
pHW-R	CTCCCCCCCAACTTCGGA

### Rescue of lentiviruses expressing the NA protein

293T cells were seeded in 100 mm cell culture dishes 1 day prior to transfection. Lentiviral expression plasmids with lentiviral packaging plasmids pLOV-CMV-NA-PuroR, pSPAX2, and pMD2G were transfected into 293T cells using PEI transfection reagent (Beyotime Biotechnology, Shanghai, China), according to the manufacturer’s instructions. The cells were incubated at 37 °C for 6 h. The supernatants were then discarded, and fresh DMEM containing 2% FBS was added. The cells were returned to the incubator and the supernatants were collected 48 h post-transfection. The collected supernatants were filtered using a 0.22 μm filter, and the lentivirus was concentrated using a Lentivirus Concentration Kit (Betoyime Biotechnology), following the manufacturer’s instructions, to obtain Lentivirus the Concentrate NA-bsd. The concentrate was stored at −80 °C.

### Construction of MDCK cell lines stably expressing the NA protein

MDCK cells were cultured in 6-well plates using a gradient of puromycin in the medium to determine the optimal concentration 1 day prior to lentiviral infection, and infection was initiated when growth reached a density of 70–80%. DMEM containing NA-bsd virus with a final concentration of 8 μg/mL polybrene was added to the MDCK cells and centrifuged at 1500 rpm for 1 h at room temperature, followed by incubation for 8–12 h. The cells were then transferred to DMEM containing 2% FBS for further incubation. After 48 h, the supernatant was discarded, the optimal concentration of puromycin-containing DMEM was added, and the cells were further cultured for 48 h. Screening with puromycin was repeated three times to obtain MDCK cells capable of expressing NA. Total cellular proteins were collected for western blotting to verify the expression of the NA protein, and the positive cells were named NA-MDCK. Positive cells were subcloned using the limited dilution method, and this was repeated three times to obtain monoclonal NA-MDCK cells. Each subclone was subjected to western blotting to verify NA protein expression.

After obtaining NA-MDCK cell line stably expressing NA, NA-MDCK were inoculated into 6-well cell culture plate 1 day in advance, and on the second day the culture medium was changed to DMEM containing 1% BSA, 1 μg/mL TPCK-treated trypsin, and incubated at 37 °C for 24 h. The plates was then removed and the medium supernatant was collected and centrifuged to remove impurities such as cell debris to prepare protein samples; the cells in the culture dishes were washed three times with PBS and then lysed by RIPA to prepare total cellular proteins. Negative control protein samples were prepared using MDCK as described above, and all protein samples were subjected to western blotting to check whether the NA of NA-MDCK was simultaneously secreted into the cell culture medium.

### Construction of recombinant strains carrying two H9N2 AIV HA genes

Six bidirectional expression plasmids (pHW-PR8-PB1, pHW-PR8-PB2, pHW-PR8-PA, pHW-PR8-NP, pHW-PR8-NS, and pHW-PR8-M) from A/Puerto Rico/8/34/H1N1 (PR8) were used as the backbone, and the remaining two bidirectional expression plasmids, pHW-PR8-HA and pHW-PR8-NA, were replaced with the recombinant plasmids pHW-SD18HA and pHW-PR8NAPS-GD12HA.

293T cells were seeded into 6-well culture plates, and transfection was initiated when their growth density reached 70–80% confluency. The above plasmids were transfected into 293T cells using the Lipofectamine2000 transfection reagent (Thermal Fisher Scientific, Waltham, Massachusetts, USA) according to the manufacturer’s instructions. After 4 h of transfection, the culture medium of each well was replaced with DMEM containing 1% bovine serum albumin (BSA), 1 μg/mL TPCK trypsin, and 20 mU/mL NA protein. The cells were then incubated for an additional 48 h. Subsequently, the entire cell culture plate was placed in a −80 °C freezer and thawed on ice after being completely frozen three times. The thawed liquid was then collected and centrifuged at 4 °C and 1500 rpm for 10 min. The supernatant was carefully collected for further use.

NA-MDCK cells were spread onto 100 mm cell culture dishes and inoculated with the recombinant virus after the cell density reached 80%–90%. The supernatant containing the recombinant virus was diluted 1000-fold with DMEM containing 1 μg/mL TPCK trypsin and then inoculated onto the NA-MDCK cells. After 72 h, the entire dish was placed into a −80 °C refrigerator and thawed on ice after being completely frozen three times. The supernatant was collected in a freeze-thawed solution at 4 °C and stored separately at −80 °C. The rescued virus (H9-SD18GD12HA) was passaged on NA-MDCK cells, and hemagglutination was measured. Simultaneously, RNA was extracted from the culture medium supernatant and subjected to reverse transcription PCR and sequencing by gel recovery. The collected medium supernatant containing H9-SD18GD12HA continued to be cultured on NA-MDCK cells until the 10^th^ generation and was stored at −80 °C for subsequent experiments.

### Determination of viral biological characteristics

The supernatant of the medium containing H9-SD18GD12HA was diluted 1000-fold and inoculated into normal MDCK and NA-MDCK cells for 72 h. The supernatants were collected, and the hemagglutination potency of the supernatant cultures was determined using a hemagglutination assay.

The collected virus-containing allantoic fluid was diluted tenfold with PBS, and each dilution of the allantoic fluid was inoculated into 9–11-day-old SPF chicken embryos through the allantoic cavity. Embryonated-eggs with embryos that died within 24 h after inoculation were discarded, and allantoic fluid was collected from the remaining embryonated-eggs after 96 h of incubation. The hemagglutination potency of the allantoic fluid from the embryos at each dilution was determined. Hemagglutination efficiency was calculated according to the Reed–Muench method as the median egg infectious dose (EID_50_).

The supernatant of cells containing recombinant virus was diluted tenfold with DMEM containing 1% BSA, 1 μg/mL TPCK-treated trypsin, and streptomycin-penicillin (double antibiotic). Each dilution of the viral solution was inoculated into NA-MDCK cells, which were cultured in a cell culture incubator with 5% CO_2_ at 37 °C for 72 h. The cytopathic effect was observed and the median tissue culture infectious dose (TCID_50_) was calculated according to the Reed–Muench method.

After inoculating NA-MDCK cells with recombinant and parental viruses at a dose of 1 multiplicity of infection (MOI), the TCID_50_ at different time points was determined, and growth kinetic curves were plotted.

### Virus shedding and antibody level testing in chicken

To verify the immunological effect of H9-SD18GD12HA as an antigen in chicken, two groups of 3-week-old SPF chickens (*n* = 23) were separately vaccinated with H9-SD18GD12HA or PBS buffer as the main ingredient in the vaccine using eye and nose drops. Specifically, using a 200 μL manual pipette as an alternative to a dropper bottle, 200 μL of vaccine solution or PBS buffer was aspirated one at a time, and half of the liquid was added to the eye, with the remainder dripping in through one side of nostril. It is important to note that the head of the chicken should be fixed and the other nostril temporarily blocked during immunisation to ensure full absorption of the fluid. The dose of H9-SD18GD12HA was 200 μL per chicken (10^5^ TCID_50_/200 μL). Oropharyngeal and cloacal swabs were collected at 3, 5, and 7 days post-immunization (dpi) and stored in double-antibiotic PBS. SPF chick embryos at 9–11 days of age were inoculated at 0.2 mL per embryo, and urothelial fluid was collected 72 h after inoculation. Virus shedding was determined by measuring the hemagglutination potency using a hemagglutination assay.

Serum antibody levels were determined using the hemagglutination inhibition assay. Venous blood samples were collected at 7, 14, and 21 dpi. Serum was separated and reacted with four-unit antigens, GD12 and SD18, to determine the hemagglutination inhibition potency.

The expression level of bronchial mucosal IgA was determined by ELISA. Three chickens from each of the two groups of SPF chickens were euthanized at 3, 7, and 21 dpi. The trachea was then collected and rinsed with PBS. The lavage fluid was collected, and its IgA level was determined according to the instructions of the Chicken IgA ELISA assay kit (Beyotime Biotechnology).

### Detection of cytokine transcript levels by fluorescence quantitative PCR

One chicken from the H9-SD18GD12HA live vaccine group and one from the PBS group were sacrificed at 3, 7, and 21 dpi. The trachea, lungs, and spleen were collected, and a small amount of tissue was clipped with scissors for grinding. After grinding, the tissue was put into a centrifuge at 3500 rpm for 10 min at 4 °C, and the supernatant was aspirated for RNA extraction and determination of RNA concentration. The RNA obtained was reverse transcribed to cDNA, and the transcript levels of interleukin (IL)-1β, IL6, and interferon (IFN)α were detected by fluorescence quantitative PCR (qPCR).

### Detection of immunological effects

Since H9N2-infected chickens usually have only mild symptoms or no disease, the evaluation of the effectiveness of the H9N2 vaccine is more concerned with whether the vaccine can reduce or even prevent viral shedding after the attack. After 21 days of immunization, the H9-SD18GD12HA vaccinated group was randomly and equally divided into two groups, A and B. The PBS control group was randomly divided into two groups, C and D. Groups A and C were attacked by the GD12 strain, while groups B and D were attacked by SD18 strain, and the dose were both 10^6^ EID_50_/0.2 mL. Oropharyngeal swabs and cloacal swabs were collected at 3, 5, 7, 9 days post-challenge and inoculated with SPF chicken embryos. After 72 h of inoculation, the urothelial fluid was collected to determine the virus shedding by measuring the hemagglutination potency.

### Statistical analysis

Data presentation and statistical analyses were performed using GraphPad Prism (version 8.0). The data are presented as the mean fold change ± SD. Statistical significance was determined using a Student’s *t*-test for pairwise comparisons. Statistical significance was set at *P < 0.05, **P < 0.01, and ***P < 0.001.

## Results

### Genetic and evolutionary traits

**Figure 1:**
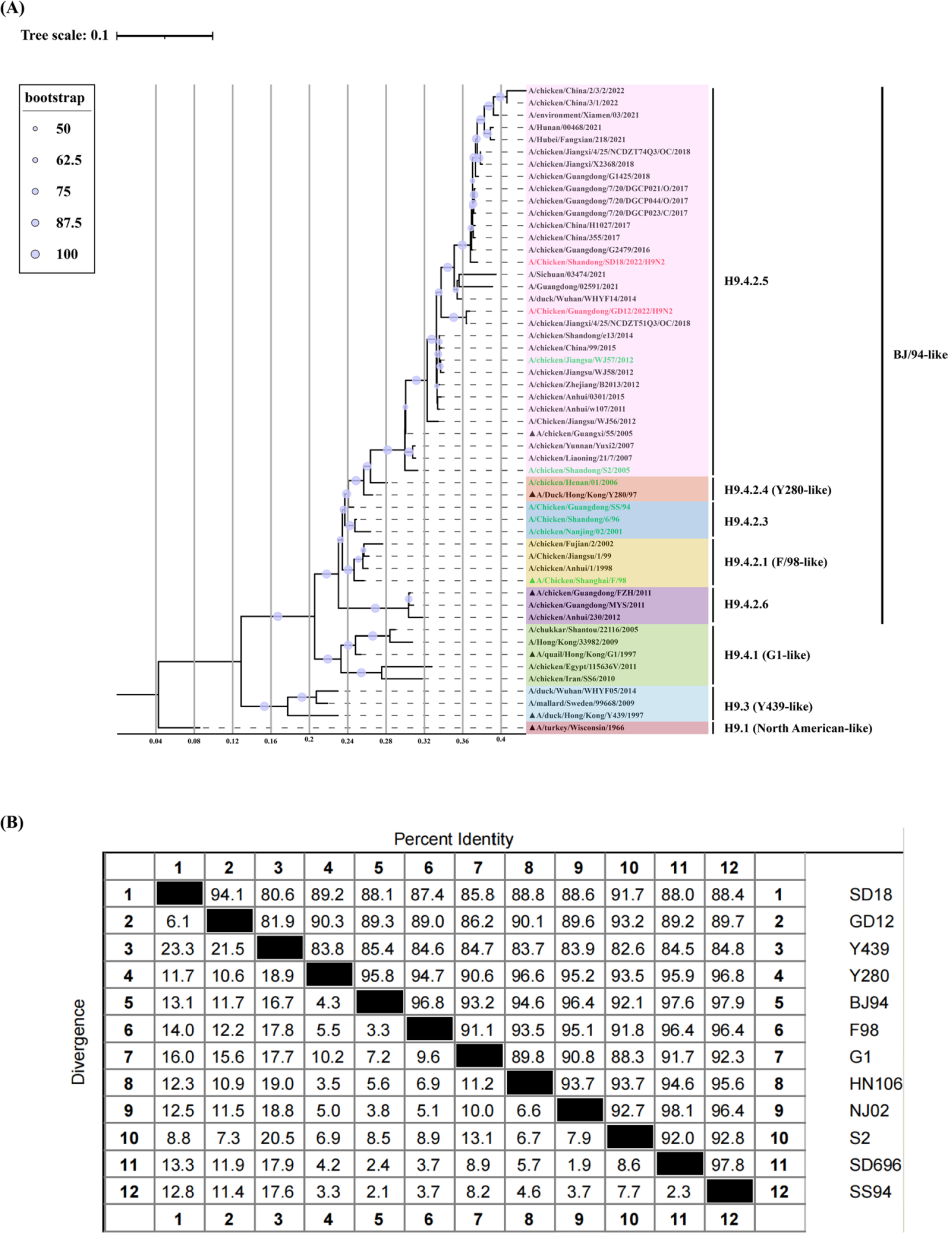
**Phylogenetic tree and nucleotide identity comparison results.** **A** The Phylogenetic tree of the HA gene of H9N2 subtype AIV was generated using Phylosuite, and the sequences were obtained from NCBI and GISAID EpiFlu databases. The generated phylogenetic tree was visualized using iTOL. The sequences marked in red are the strains isolated in Guangdong and Shandong Province in this study; sequences labeled in green are vaccine reference strains; sequences labeled using black up-pointing triangle are classical isolates. **B** The nucleotide identity of the HA gene nucleotide sequences of the SD18 and GD12 strains were compared with those of classical strains of the Eurasian lineage and some vaccine reference strains, and the results showed that the nucleotide identity between SD18 and GD12 reached 94.1%. The classical isolate with the highest identity to both strains was Y280, with an identity of 89.2% and 90.3%, respectively.

To investigate the evolution and genetic characteristics of GD12 and SD18, we conducted a phylogenetic analysis of the full-length ORF of the HA gene for both strains. The resulting phylogenetic tree (Figure 1A), along with nucleotide identity analyses (Figure 1B), revealed that the two H9N2 subtype AIV strains belong to the h9.4.2.5 clade, a subpopulation currently prevalent in China. Additionally, the genetic distances between the two strains and the classical isolate A/chicken/Shandong/S2/2005 (S2) were relatively distant, suggesting that the viruses are still under constant mutation. In addition, commonly used commercial vaccine strains, except for A/Chicken/Jiangsu/WJ57/2012 (WJ57), were genetically distant from GD12 and SD18. Nucleotide identity between the classical isolates of each lineage and GD12 and SD18 was approximately 90%.

Potential glycosylation sites of reference strains with SD18 and GD12 were analyzed using the glycosylation site online analysis website NetNGlyc [31]. As shown in Table 3, SD18 and GD12 had 8 potential glycosylation sites, which were located at 29aa–31aa, 82aa–84aa, 141aa–143aa, 298aa–300aa, 305aa–307aa, 313aa–315aa, 492aa–494aa, 551aa–553aa, and the 2 strains had 6 stable glycosylation sites, NST at 29aa–31aa, NPS at 82aa–83aa, NVS at 141aa–143aa, NTT at 298aa–300aa, NVS at 305aa–307aa, NGT at 492aa–494aa, NGS at 551aa–553aa. At position 218aa–220aa position, both isolates lacked a glycosylation site, which was the same as the vaccine reference strains NJ02 and SD696; at 313aa–315aa, both strains added a glycosylation site, both of them NCS, which only appeared in NJ02 and S2.

**Table 3:** **Potential glycosylation sites of HA**

Strain	Potential glycosylation sites								
	29	82	141	218	298	305	313	492	551
GD12	NST	NPS	NVS		NTT	NVS	NCS	NGT	NGS
SD18	NST	NPS	NVS		NTT	NVS	NCS	NGT	NGS
Y439	NST	NPS	NVS	NRT	NTT	NVS		NGT	NGS
F98	NST	NPS	NVS	NRT	NTT	NVS		NGT	NGS
G1	NST	NPS	NVT	NRT	NST	NIS		NGT	NGS
HN106	NST	NPS	NVS	NRT		NVS		NGT	NGS
NJ02	NST	NPS	NVS		NTT	NVS	NCS	NGT	NGS
Y280	NST	NPS	NVS	NRT	NTT	NVS		NGT	
BJ94	NST	NPS	NVT	NRT	NTT	NVS		NGT	NGS
S2	NST	NPS	NVS	NRT	NTT	NVS	NCS	NGT	NGS
SS94	NST	NPS	NVS	NRT	NTT	NVS		NGT	NGS
SD696	NST	NPS	NVS		NTT	NVS		NGT	NGS

### Successful construction of MDCK cell lines capable of stably expressing NA proteins

Using the NA gene of SD18 strain as a template, the ORF of the NA gene was successfully amplified, with the size of 1457 bp. The linearized full-length fragment of pLOV-CMV-PuroR was successfully amplified to 7699 bp. Fragments of the NA ORF and the linearized vector were homologously recombined. Recombinant vector was sequenced correctly and named pLOV-CMV-NA-PuroR. Lentiviral concentration was obtained by co-transfecting the lentiviral packaging plasmid (pLOV-CMV-NA-PuroR) with helper plasmids (pSPAX2 and pMD2G) into 293T cells to obtain the lentiviral concentrate NA-bsd.

**Figure 2:**
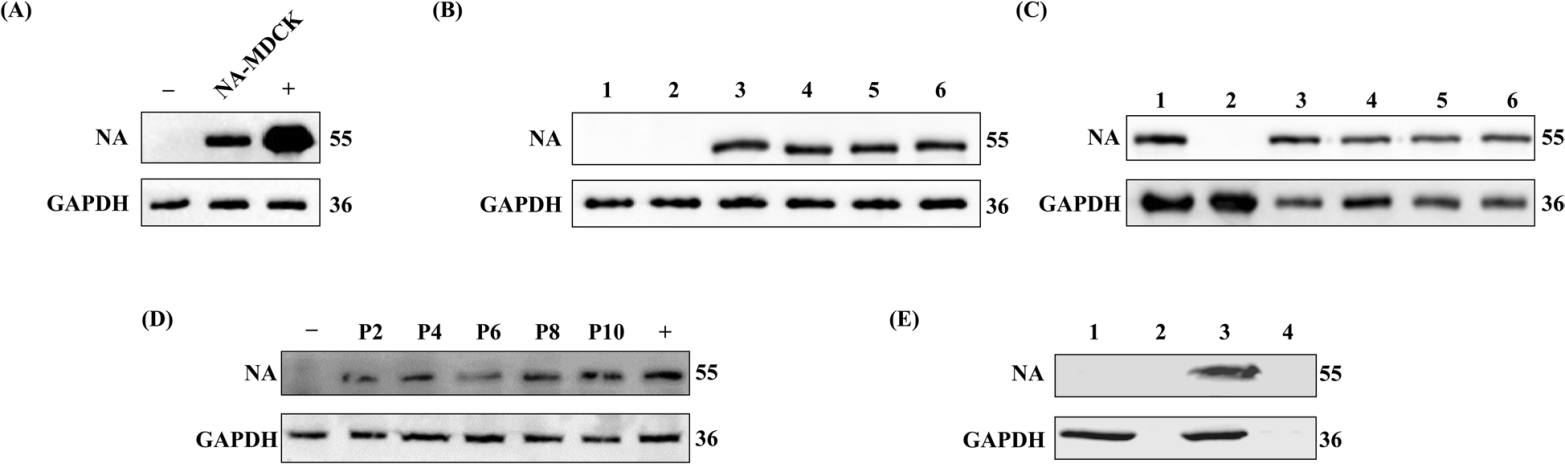
**Construction of MDCK cell lines stably expressing NA proteins.** **A** NA protein expression results of MDCK cells after puromycin screening. From left to right: negative control, MDCK cells after three puromycin screens for NA protein samples, and MDCK cells after transfection of NA eukaryotic expression plasmid as positive control. **B** The results of NA protein expression of cells in each well after the first round of subcloning. First and second well cells had no NA protein expression, cells in the third to sixth wells were able to express NA proteins normally, and MDCK cells corresponding to the fourth well were collected for the second round of subcloning. **C** The results of NA protein expression of cells in each well after the second round of subcloning. All wells exhibited normal NA protein expression except for the cells in the second well, which did not show NA protein expression. The MDCK cells from the first well were selected for serial passaging. **D** Results of protein expression stability assay of MDCK cells. From left to right: negative control, 2–5 represent MDCK cells of P2, P4, P6, P8, and P10 generations, respectively, all of which express NA protein normally, and positive control. **E** Results of western blot to verify the location of NA protein expression of NA-MDCK. From left to right: MDCK cell protein sample, MDCK medium supernatant, NA-MDCK cell protein sample, NA-MDCK medium supernatant.

MDCK cells obtained by lentiviral infection were screened using puromycin to obtain cells capable of expressing NA proteins. After three rounds of puromycin screening, some cells were collected and normal MDCK cells were used as a negative control, and the eukaryotic expression plasmid carrying the NA gene was transfected into MDCK cells as a positive control. The results were shown in Figure 2A, the control and experimental groups had the same concentration of GAPDH protein expression at 30 kDa, and no band was seen in the negative control group at 55 kDa, whereas a clear band could be seen in the positive control group, NA-MDCK group, indicating that the lentiviral infection was successful and the NA protein was successfully expressed. After two rounds of subcloning, one strain with a better protein expression effect was screened and successively passed on for 10 generations (Figures 2B and C). We found that the NA-1 strain in the second round of subcloning was capable of expressing the NA protein in the 10^th^ generation (Figure 2D); therefore, this strain was named NA-MDCK and was used for subsequent experiments. The result of western blot (Figure 2E) indicates that NA-MDCK-expressed NA protein is mainly cell-derived and is not secreted into the cell culture medium.

### Successful construction of recombinant viruses expressing HA proteins of two strains simultaneously

**Figure 3:**
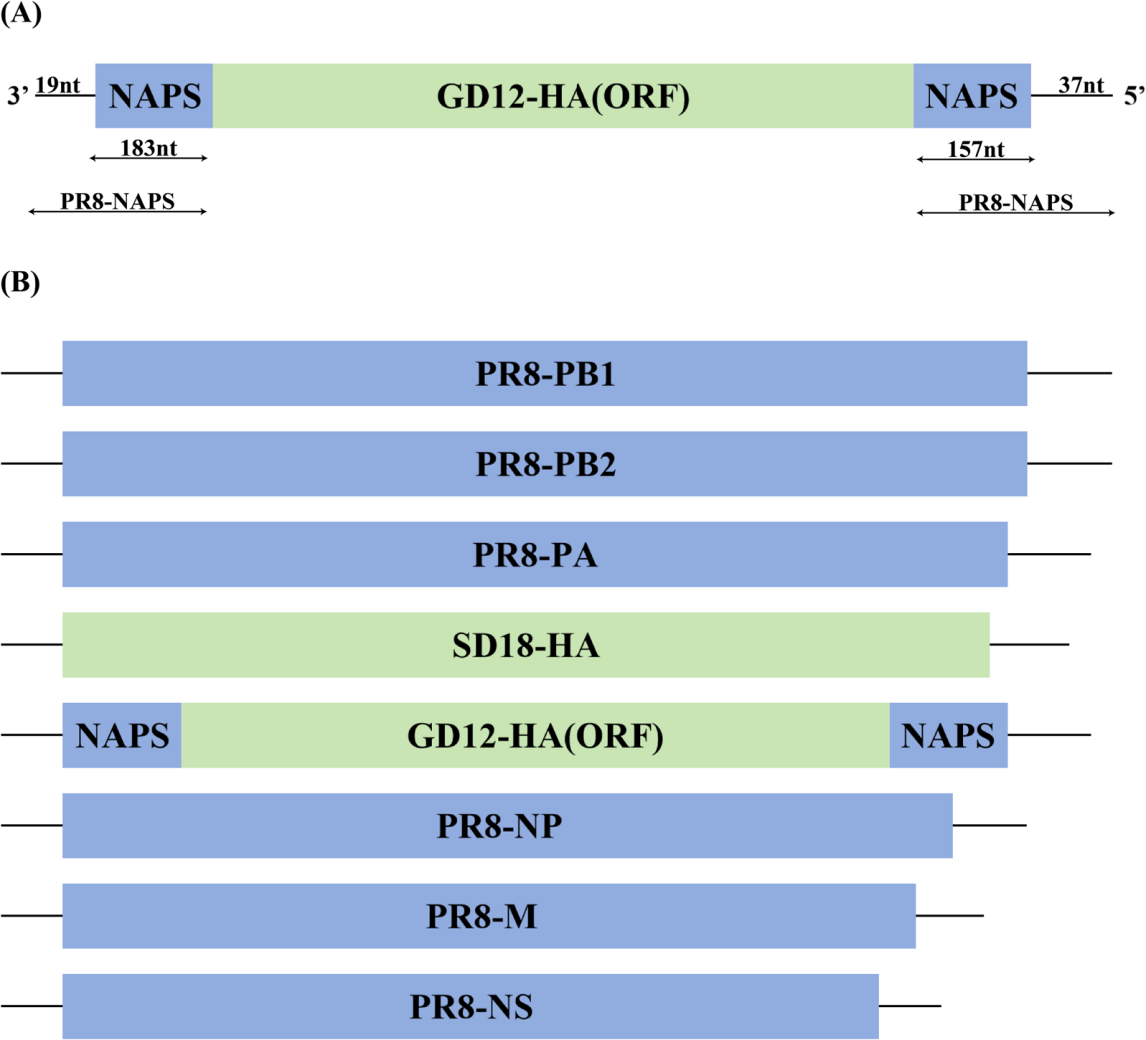
**Schematic representation of the gene structure of the recombinant virus.** The 8-plasmid reverse genetic system of the PR8 strain was used as a template, and the HA gene of the PR8 strain was replaced with the HA gene of the SD12 strain. The NA gene of the PR8 strain was retained for the packaging signal, where the ORF was replaced with the ORF of the HA gene of the GD12 strain.

**Figure 4:**
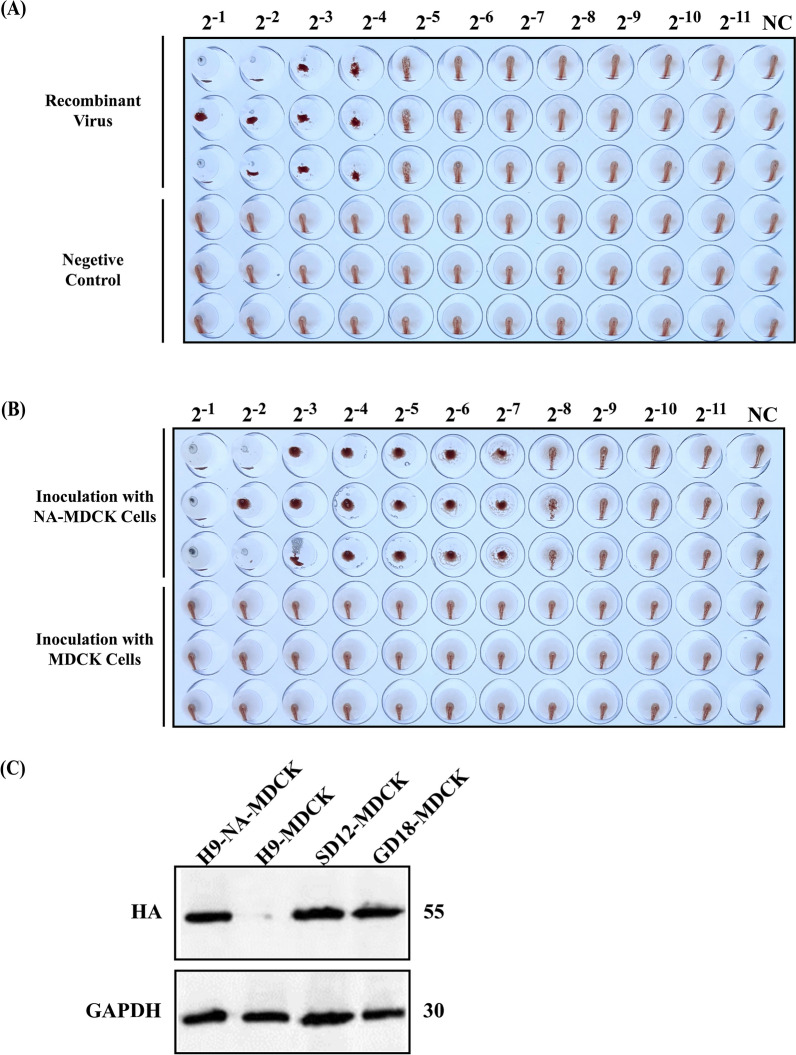
**Rescue and characterization of H9-SD18GD12HA.** **A** The HA test results for the rescued strain showed successful rescue and release of H9-SD18GD12HA into the supernatant after 72 h of incubation, as evidenced by hemagglutination of the culture medium supernatant. **B** When H9-SD18GD12HA was diluted and inoculated into both NA-MDCK and MDCK cells simultaneously, hemagglutination was observed in the supernatant of the NA-MDCK group but not in the MDCK group. **C** Western blot analysis of protein samples prepared from inoculated MDCK and NA-MDCK cells demonstrated that H9-SD18GD12HA, lacking the NA gene, could not proliferate in normal MDCK cells. However, it was able to proliferate normally in NA-MDCK cells. From left to right: H9-SD18GD12HA inoculated with NA-MDCK cells, H9-SD18GD12HA inoculated with MDCK cells, SD12 inoculated with MDCK, and GD18 inoculated with MDCK.

The recombinant plasmid constructed according to the scheme shown in Figure 3 was transfected into 293T cells and inoculated into stably passaged NA-MDCK cells. We found that the collected supernatant was able to agglutinate chicken erythrocytes (Figure 4A). The culture medium supernatant was sequenced correctly, which verified that the sequence of NA gene ORF of GD12 was correct, proving that the recombinant virus H9-SD18GD12HA was successfully rescued and the NA gene ORF of GD12 was able to be encapsidated into the virus.

After inoculation of H9-SD18GD12HA into NA-MDCK and MDCK cells, no hemagglutination was observed in the supernatant of the MDCK group, whereas hemagglutination was detected in the NA-MDCK group (Figure 4B), indicating that the recombinant strain was unable to proliferate in normal MDCK cells because of the absence of the NA gene, whereas the NA protein expressed by NA-MDCK cells assisted the recombinant strain to grow normally. The result of western blotting (Figure 4C) showed that after the deletion of the NA gene, H9-SD18GD12HA was unable to propagate normally, whereas it was able to propagate normally in NA-MDCK cells, similar to its parental strains SD12 and GD18.

### Biological characterization of recombinant strains

Results in Table 4 indicated that H9-SD18GD12HA had a lower proliferative capacity in NA-MDCK cells than parental strains.

**Table 4:** **TCID**_**50**_** and HA potency of recombinant and parental strains**

Strain	TCID_50_ (0.1 mL)	HA (log_2_)
GD12	10^–6.15^	8
SD18	10^–6.07^	9
H9-SD18GD12HA	10^–5.13^	6

**Figure 5:**
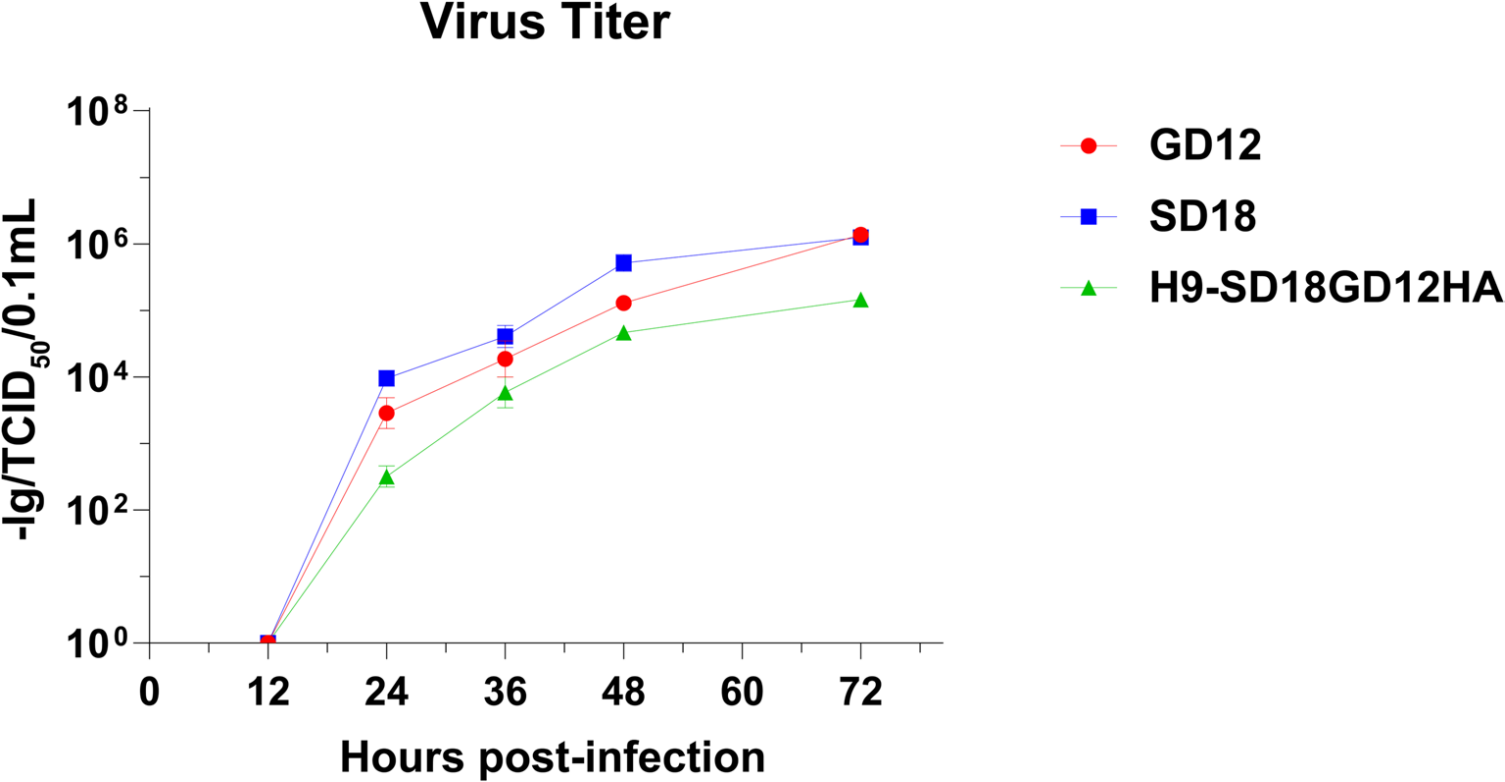
**Growth kinetic curves of H9-SD18GD12HA and two parental strains**.

The results in Figure 5 demonstrated that the TCID_50_ of the parental strains SD18 and GD12 showed a rising trend before 48 h, and the rising trend was relatively smooth at 48–72 h. Compared to the parental strains, the TCID_50_ of H9-SD18GD12HA increased more slowly, and the peak titer was lower than that of the two parental strains.

### Effectiveness of live vaccines prepared from recombinant strains in protecting SPF chickens

No viral shedding was detected in oropharyngeal or cloacal swab samples of 3-week-old SPF chickens within 7 days of immunization with vaccines prepared from H9-SD18GD12HA (Table 5).

**Table 5:** **Results of virus shedding of oropharyngeal and cloacal swabs after 1 week of immunization**

Inoculum	Amount	3d		5d		7d	
		O	C	O	C	O	C
H9-SD18GD12HA	23	0/23	0/23	0/20	0/20	0/20	0/20
PBS	23	0/23	0/23	0/20	0/20	0/20	0/20

O, oropharyngeal swab; C, cloacal swab

**Figure 6:**
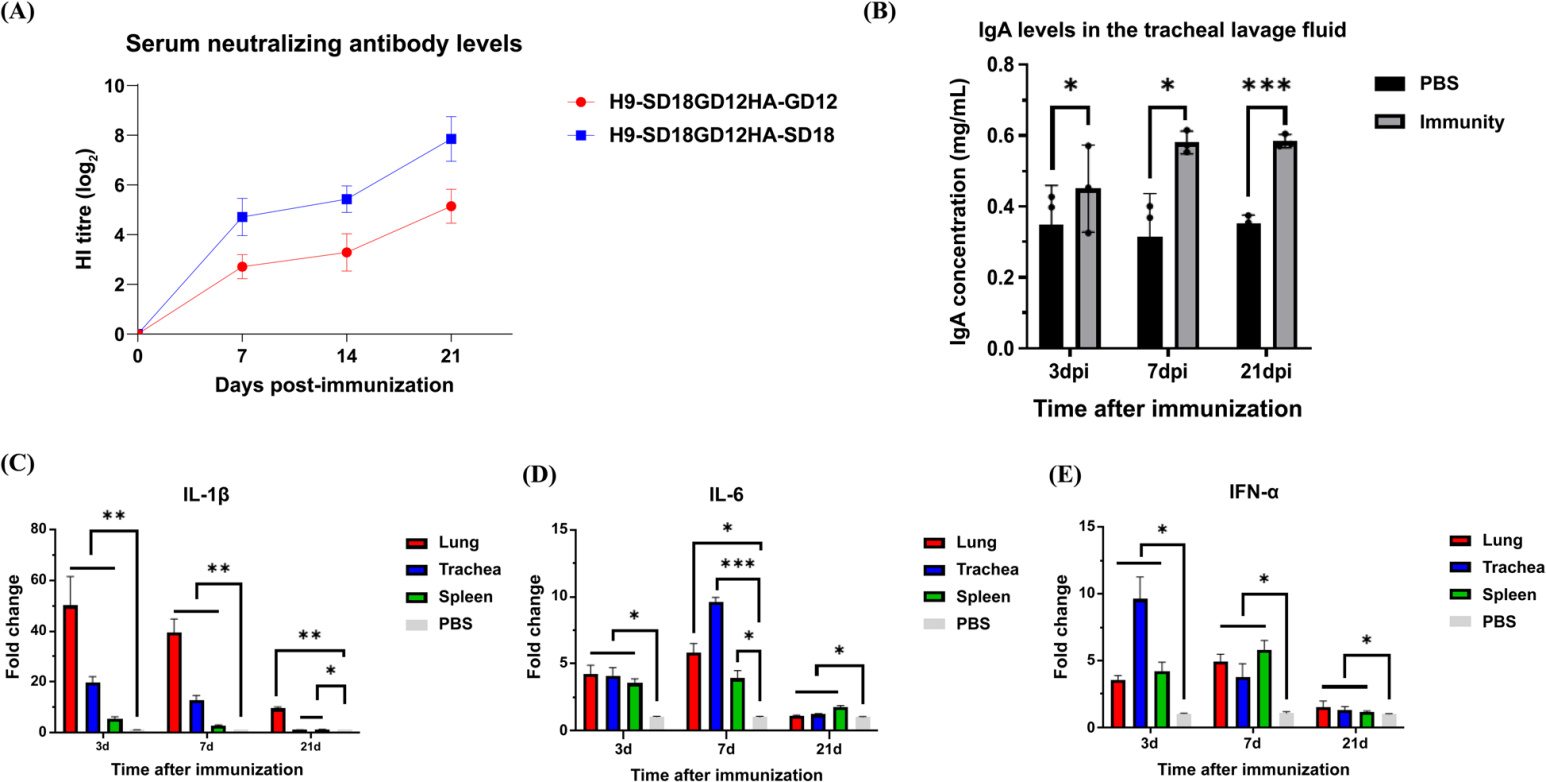
**Immunization effect of the recombinant strain-prepared vaccine reflected by detecting the serum neutralizing antibody level and the transcript levels of IL-1β, IL-6, and IFN-α in various organs after immunization.** **A** Blood was collected from all chickens at 7, 14, and 21 dpi, and the serum was separated and subjected to HI assay with GD12 and SD18 strains. **B** Three chickens from the immunized and PBS control groups were euthanized at 3, 7, and 21 dpi, and tracheal lavage fluid was collected and tested for IgA levels using an ELISA kit to assess mucosal immunization. **C**–**E** Three chickens in the immunized and PBS control groups were euthanized at 3, 7, and 21 dpi, and lung, trachea, and spleen tissues were collected and tested for IL-1β, IL-6, and IFN-α transcript levels by qPCR to assess the level of cellular immunity. The data are presented as the mean fold change ± SD. *P < 0.05, **P < 0.01, and ***P < 0.001, compared to the values of the PBS group.

The HI test on serum collected at 7, 14, and 21 dpi showed that the hemagglutination inhibitory potency of immunized chickens against SD18 was higher than that against GD12, reaching 4.25 log_2_ at week 1 and increased to 8.15 log_2_ at week 3, whereas the hemagglutination inhibitory potency of GD12 reached 5.13 log_2_ only at week 3 after immunization (Figure 6A).

The IgA expression level in the tracheal lavage fluid was detected by ELISA, and the results showed that the IgA expression level in the respiratory mucosa of the immunized group was considerably higher than that in the PBS group at days 3, 7, and 21 after immunization. Moreover, the IgA expression level gradually increased with time following immunization and was maintained at the level of 0.55 mg/mL on the 7^th^ and 21^st^ days. However, no significant change was observed in the IgA levels in the PBS group (Figures 6B and C).

The cellular immunity level of the vaccines was evaluated by fluorescence quantitative PCR to detect the cytokine transcript levels of IL-1β, IL-6, and IFN-α in the trachea, lung, and spleen of chickens at days 3, 7, and 21 after immunization. Compared with the PBS group, the transcript levels of IL-1β in the three organs peaked at day 3, followed by a decline at days 7 and 21. On day 3, the highest IL-1β transcript levels were found in the lungs, followed by the trachea, reaching approximately 19 times the levels in the PBS group. The spleen had the lowest transcript levels, at about fivefold (Figure 6C). On day 7, IL-1β transcript levels began to decline and were at their lowest in all organs by 21^st^ day, when IL-1β transcript levels were ninefold in the lungs and only 1.3-fold in the trachea and spleen in the PBS group (Figure 6C). The IL-6 transcript levels of the organs did not show significant differences between the two groups at day 3, and all of them showed an approximately threefold compared with the PBS group. The highest IL-6 transcript level of the trachea reached ninefold at day 7, while the IL-6 transcript levels of all three organs fell back to approximately onefold at day 21. IL-6 transcript levels in the lungs and spleen were 9- and 1.3-fold, respectively, in the PBS group (Figure 6D). Compared with the PBS group, IFN-α transcript levels in the lungs and spleen showed the same trend, reaching the highest level at day 7, whereas the trachea reached the highest level on day 3, followed by a gradual decline. On day 21, IFN-α transcript levels in the three organs decreased to onefold more than that in the PBS group (Figure 6E).

**Figure 7:**
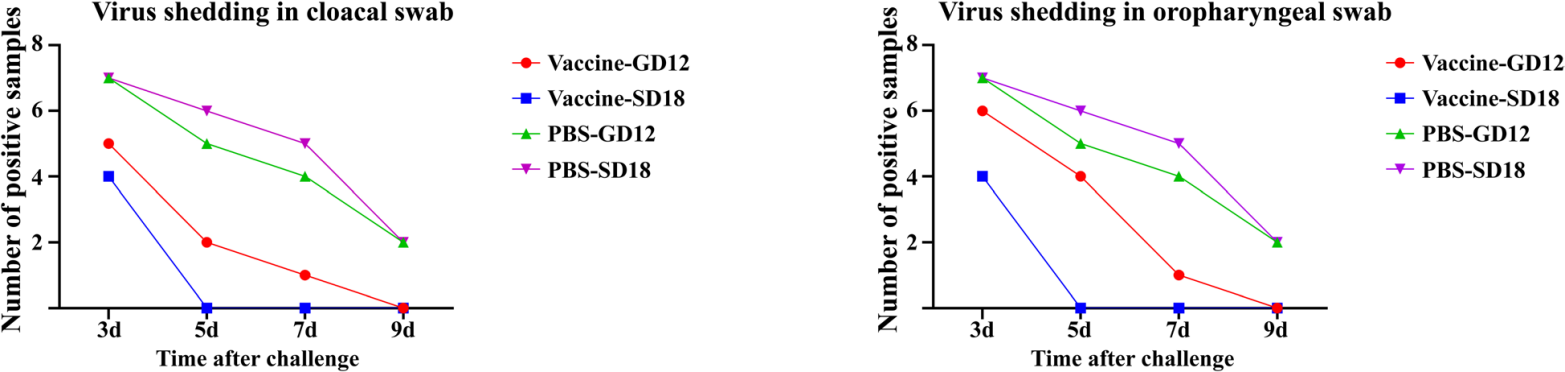
**Detection of virus shedding after virus challenging.** Oropharyngeal and cloacal swabs from the immunized and control groups were collected at 3, 5, 7, and 9 days after challenge and inoculated with SPF chicken embryos to determine the virus shedding.

We collected oropharyngeal and cloacal swabs on day 3, 5, 7, and 9 after the challenge and detected viral shedding using hemagglutination test (Figure 7). The test results showed that after vaccination with the full-length HA of SD18, both oropharyngeal and cloacal swabs showed no virus shedding at 5 days, while the PBS group continued to show positive results until day 9. Virus shedding in the GD12 strain was no longer present on day 9. Compared with SD18, this vaccine is weaker in inhibiting virus shedding on GD12 infection, but it can still play a role in advancing the time window for the cessation of virus shedding.

## Discussion

Currently, the prevention and control of H9N2 AIV in China is mainly carried out through the injection of inactivated vaccines; however, due to the disadvantages of older vaccine reference strains and the difficulty of inactivated vaccines in inducing cellular immunity, the prevention and control of H9N2 in recent years has been relatively ineffective, and it has been impossible to solve the problem of long-term carryover of the virus and virus shedding of H9N2. Considering H9N2’s contribution to other subtypes of influenza variants and its public health hazards, the development of novel vaccines that can effectively control H9N2 infection is of interest.

We successfully isolated two H9N2 subtype AIV strains, SD18 and GD12, from samples collected during the epidemiological survey, and it was clear from genetic evolutionary analyses that the two newly isolated strains were not genetically distant from each other, even though they were collected from different provinces in China, implying that they may be mutated from the same strain. In terms of their positions in the phylogenetic tree, the H9N2 strains isolated in the last 2 years (including SD18 and GD12) are genetically distant from the classical reference and vaccine strains and are not part of the same subpopulation. Therefore, undoubtedly the marketed vaccines are unable keep up with the evolutionary speed of H9N2. Moreover, a vaccine using these two strains as templates would be able to effectively prevent and control H9N2 or at least slow down its evolution. To broaden the scope of the vaccine, enhance its immune effect, and minimize virus shedding, we prepared a live vaccine to provide a better immune effect and cope with the rapid evolution of H9N2.

HA and NA are two glycoproteins on the surface of AIV [32], and these two proteins play different and important roles in the replication cycle of the virus. The HA protein acts mainly in the pre-infective phase of the virus by initiating endocytosis through binding to the salivary acid receptor on the surface of the host cell and eventually undergoes membrane fusion to allow the entry of whole virus particles into the cell [33]. The NA protein functions mainly in the process of viral outgrowth and releases into the extracellular space, facilitating the release of the virus from the infected cell by cleaving the terminal sialic acid residues that are present on the surface of the cell and in the progeny virus particles, thereby playing an important role in the release and propagation of the progeny virus particles [34].

Since the establishment of the reverse genetic system of the influenza virus in 1999, it has been widely used in the development of influenza vaccines. The 8-plasmid viral rescue system is capable of complete viral reconstruction and contains bidirectional expression plasmids capable of expressing PB2, PB1, PA, HA, NP, M, NS, and NA proteins [18]. Here, we used the 8-plasmid influenza reverse genetics system with the PR8 strain as the backbone to construct the H9-SD18GD12HA virus. This involved replacing the HA fragment in the HA-expressing vector with the intact HA fragment of SD18. In the NA-expressing vector, the NA packaging signal was retained, while the ORF was replaced with the HA ORF from GD12. Given that the NA protein is an important capsid protein of the influenza virus, the replaced protein can be displayed on the surface of the virus which can stimulate the body to generate an immune response for the production of corresponding antibodies [35]. Simultaneously, since the NA protein not only binds to the receptor but also plays an important role in the viral budding process [36–38], the replacement of H9-SD18GD12HA with the NA protein ORF results in the loss of viral outgrowth, signifying that even if H9-SD18GD12HA invades the cell and completes genome replication, it will not be able to outgrow the virus and complete the viral reproduction process. The recombinant virus constructed based on the above idea can display the HA protein of two strains (SD18 and GD12) on the surface of the virus and cannot propagate. Accordingly, we constructed an NA-MDCK cell line which is able to express the NA protein, thereby assisting viral propagation under controlled conditions.

Furthermore, the utilization of PR8 as a backbone may enhance the pathogenicity of the virus in mammals. However, the absence of NA in H9-SD18GD12HA hinders its capacity for effective replication in mammals (e.g., humans), thereby mitigating the potential for mammalian adaptation of the PR8 backbone. Recent studies have shown that by rearranging some internal genes and making additional modifications to HA and NA, the ability to transmit recombinant strains can be reduced and reassortment can be avoided while inducing effective immunity in vivo [26]. This has given us some inspiration and we will be combining these ideas in further research to try and optimize the reassortment risk from the strain backbone as much as possible, while maintaining excellent immunity.

It is worth noting that the HA/NA balance of H9-SD18GD12HA after gene replacement might be disturbed [39], and therefore, whether it could grow normally in susceptible cells or chicken embryos remains under consideration [40, 41]. In addition, the viral titer of H9-SD12GD18HA was lower than that of the two parental strains at all time points in the growth curve measurements. Considering that H9-SD12GD18HA was missing the NA ORF, the decrease in the proliferative capacity was more likely due to the fact that the amount of NA expressed by the NA-MDCK during viral proliferation was insufficient to support the demand for NA protein function of the virus at the peak of replication. In addition, substitution of NA ORF may also lead to disorganization of viral surface proteins, and changes in RNA secondary structure may make viral replication less efficient.

Immunization with inactivated vaccines protected chickens to some extent during the early years of H9N2’s emergence in China. However, since 2015, the H9N2 strain has experienced significant antigenic drift, which has made it difficult for existing inactivated vaccines to match prevalent strains. The inherent challenges of inactivated vaccines, such as virus shedding and difficulty in triggering cellular immunity, have not been resolved [14, 42]. It has been shown that commercial inactivated vaccines using earlier strains as reference sequences have had little effect on protecting chickens against recently prevalent wild strains in terms of symptomatic relief and reduced virus shedding [43], implying that there is an urgent need to develop vaccine classes that are more similar to the prevalent strains in order to offer better protection. The results of the SPF chicken protection test showed that the vaccine prepared by H9-SD18GD12HA was able to elicit a good immune response in chickens and induce mucosal and cellular immunity in the organism. Owing to the modification of the two surface proteins of the original viral backbone, this vaccine can also replace the two recombinant fragments with antigenic genes of other influenza virus subtypes or antigenic genes of other avian infectious disease viruses to achieve cross-protection and extend the scope of vaccine protection. Both immunological and post-attack data suggest that the complete HA sequence with SD18 inserted provides better protection than GD12 with only the HA ORF inserted. Considering that the sequence difference between the two is not large, the difference in immunological effects is more likely due to the fact that the HA ORF is not optimally compatible with NA packaging signals at both ends. The packaging signals determine the efficiency of viral replication and vRNA packaging [20]. However, HA has a much stronger preference for packaging signals [44], and the use of mismatched packaging signals is not conducive to the viral packaging of recombinant RNA fragments. It has been pointed out that the genome packaging of AIV and the RNA-RNA interactions involved present a complex and tedious process [37, 45]; therefore, the insertion of recombinant vaccines with only the ORF frame should be considered and tested to ensure that the inserted antigen can be expressed efficiently for better immunological effects. Meanwhile, the evaluation of the immunological effects of the recombinant vaccine in this study focused more on mucosal and cellular immunity, as better mucosal and cellular immunity tends to imply that the vaccine is able to induce a more durable and effective immune response against such respiratory-transmissible pathogens and reduce the duration of virus shedding. The assessment of humoral immunity in this study was mainly represented by the titre of HI in serum. In the subsequent investigation, in order to comprehensively assess the level of humoral immunity, we will consider the use of commercial avian influenza virus antibody ELISA kit to deepen the understanding of the effect of recombinant vaccine in stimulating humoral immunity [26, 28, 46].

In conclusion, we developed a replication-deficient recombinant live vaccine based on the PR8 strain as a backbone, in which we inserted the HA gene fragments of the two strains isolated in previous epidemiological investigations, and demonstrated that it could only reproduce in modified cell lines and elicit humoral and cellular immunity in chickens. As mucosal immunity and virus shedding reduction of this vaccine have great advantages over traditional inactivated vaccines, its development value and potential as a new H9N2 subtype influenza vaccine are high.

## Data Availability

All data generated or analysed during this study are included in this published article.
